# 

**Published:** 1881-03

**Authors:** 


### CONTENTS FOR MARCH, 1881.

ORIGINAL COMMUNICATIONS.

ART. I.—Some facts Concerning Human Hybridity. By HARVEY L. BYRD, M. D.... 101
ART. II.—The Proper Mode of Vaccinating. By BARTON DOZIER, M. D.... 105
ART. III.—Earthy Deposit in the Cornea. By S. J. PARKER, M. D.... 106
ART. IV.—Treatment of Fractured Jaw without teeth in the Mouth. By J. ADAMS BISHOP, M. D.... 108

CORRESPONDENCE,

Reports of Medical Society Proceedings.... 110

EDITORIAL DEPARTMENT.

Gunshot Wounds.... 112
Vaccination.... 113
A Table of Sounds.... 113
Cremation.... 115
Leprosy.... 116
Troubles of Journalism.... 116
The Physiological Antagonism of Remedies.... 117
Apocynum Cannabinum in Dropsy.... 119
Iodine in Diphtheria.... 119
Baltimore College of Dental Surgery.... 120
University of Maryland Medical School.... 120
Alumni Associations.... 120
For a Mad Dog's Bite.... 121
Goodyear Dental Vulcanite Co.... 121
Dental Journalism.... 122

BIBLIOGRAPHICAL DEPARTMENT.

Medical Diagnosis, with special reference to practical Medicine.... 124
Transactions of the American Medical Association.... 125
Transactions of the eleventh annual session of the Medical Society of Virginia.... 125
Transactions of the Mississippi State Medical Association.... 125
Report of Health Department of Baltimore, Md.. 126
Eleventh and twelfth annual reports of Maryland Eye and Ear Infirmary.... 126
Tenth annual report of the Board of Directors of the Children's Hospital of D. C.... 126
New York Medical Journal.... 126
Physician and Surgeon.... 126
Mississippi Valley Medical Monthly.... 127
A copy received of "Remarks on Syphilis.... 127
Zahnarztlicher Almanach, 1881.... 127
Ohio State Journal of Dental Science.... 127
THERAPEUTIC PEARLS AT RANDOM STRUNG....128–132
NECROLOGICAL....132–135

EXCERPTA.

Dangers of "Bonwill's Method" of Anæsthesia.. 136
International Medical Congress.... 136
About Coin.... 137
Sad end of a Promising Life.... 137
Hypnotism, Catalepsy and the faculty of speech.. 138
Longevity of Medical Men.... 140
For Arsenic Poisoning.... 141
Iodide as a specific in Croupous Pneumonia.... 141
Treatment of Pneumonia.... 141
Nitro-Glycerine in Bright's Disease.... 143
Prevention of Laceration of the female perineum. 144
Method of withdrawing fluids from the Ear.... 145
Hot Rectal Douche.... 146
How to remove Corns.... 148
Therapeutics in Nitro-Glycerine.... 149

